# Editorial and Miscellaneous

**Published:** 1861-08

**Authors:** 


### EDITORIAL.

*Rush Medical College.*—We feel authorized in saying that the prospects of this long established and well known institution, were never more flattering than at the present time. Some little question, we understand, has arisen with reference to the appointment of Professors Blaney and Freer as Brigade Surgeons in the army. To avoid any misapprehension, we venture the following statement: The gentlemen mentioned will give their usual courses of lectures this winter as heretofore. Their appointment has especial reference to the establishment in this city of a great central military depot and hospital; to which latter all invalided and wounded soldiers will be conveyed and cared for, under their immediate supervision. The opportunity for clinical observation thus afforded, it will be seen, will be of the most superior character, in fact unapproachable elsewhere.

The peculiar salubrity of Chicago, as proved by statistics and general observation, has commended it to the Department as remarkably favorable for the establishment of a general military hospital, and thus the students of Rush Medical College will be supplied with means of clinical study of the very highest order.

Arrangements have also been made by which the dispensary department of the College will prove unusually rich and instructive.

In view of the foregoing facts, the friends of the institution are requested to correct the various injurious rumors which designing enemies of this College have set afloat for purposes easily to be surmised. We find it necessary to unearth the burrowing schemes of these unscrupulous and desperate foes, not only of the College, but of all *honorable and legitimate* medical teaching.

*Ethical.*—It is not in accordance with the code, or with ordinary gentlemanly business courtesy, for a practitioner called in a case of emergency, in the absence of the regular family physician, to assume the case unless he is distinctly informed that the usual attendant is discharged.

It is unnecessary for the transient physician thus summoned to call more than half a dozen times to ascertain how the patient is getting along. Poverty is an unpleasant incident of professional life, but scheming dishonesty and parasitic fawning and fulsome lavishing of unsought attention, are worse.

It is wholly needless for a man on donning the M. D. to slough off the gentleman. If the snake or other reptile predominates in your constitution, for heaven's sake put on a better looking skin when you shed the old one—code or no code.

---

*The State Examining Board—Eight Dollars a Day, &c.*—Nothing conduces to industry like good pay. Under the influence of this potent stimulus, or from a desire to "elevate the profession," the new Examining Board still continues its labors. Two candidates *per diem* emerge from their green room, either adorned with military feathers or "plucked," as the case may be. It is understood that large attention is paid to the sphenoid bone and nervous ganglia and plexuses; meanwhile one of the elect has signalized his advent to the medical staff of the grand army by anointing and poulticing a femoral hemia, mistaken for a bubo. It is God's mercy he did not put a lancet into it. It is understood he—(not the bubo or the surgeon,) but the patient—has his discharge from the army.

Seriously, is not this gross professional humbug about played out? The profession is to be congratulated that quite a number of well qualified and experienced gentlemen have been permitted by the Board to go into the service. *O si sic omnes!*

*Shoulder Straps.*—An angry controversy is raging which threatens to assume stupendous proportions. The majority of the newly appointed surgeons to the volunteer regiments have donned the shoulder straps of the *Majority.* To this the Medical Bureau of the regular service stoutly objects. The United States laws upon the subject are understood to place all officers in the volunteer service on a level in rank with those holding similar designations in the regular service, and bating the usual precedence of seniority, entitled to the same pay and emoluments. But the Medical Bureau, influenced by reasons readily to be surmised, cannot bear to have the *parvenues* of the medical volunteer corps come so nearly between the wind and their nobility. Hence they insist that a Brigade Surgeon shall rank only as a Major of Cavalry; a Regimental Surgeon as a Captain of the same, and assistants as Lieutenants. This simply degrades the volunteer surgeons below their legal peers in the regular service, and what is infinitely worse than the *hiatus valde deflendus* of shoulder "fixings"—despoils them of the pay of the superior rank.

We feel no hesitancy in saying that the volunteer surgeons will not only injure themselves, but permit a wholly unworthy slur to be cast upon the entire profession if they do not resist to the last extremity such a contemptuous encroachment on their legal rights. The pay of the medical service in the army is, in comparison with the duties involved, so pitiful that few can afford to undertake the position—certainly no gentleman of adequate ability and experience who has a family dependent upon him. That even this pittance should be illegally cut down, merits nothing but scorn in the attempt. We trust the "red tape" of the Department may not be permitted to strangle the patriotic vitality of the volunteer surgeons. Stick to your rights, Messieurs Surgeons of the army—including shoulder straps and all.

It may be received as an almost self-evident proposition that the medical practitioners of civil life, in this country, have had immensely greater experience than those who, whatever their original qualifications, have been isolated with the little squads of the regular army, during the long years of peace the country has been favored with. Self-respect, if nothing else, should prevent the former from accepting the inferior position so arrogantly in defiance of the law thrust upon them.

The officers of the army usually are gentlemen, and will favor the medical officer in insisting upon his appropriate position. Let it be done.

---

We cannot resist the inclination to insert, right here, words of well deserved praise and honor to certain volunteer army surgeons who *did not run* from the battle field. The comment suggests itself—Shall such surgeons be deprived of the pay and position of gentlemen?

"*Cowards—A contrast.*—That the men of Varian's Battery can live in New York is a disgrace to civilization. Future historians will point to the popular disregard of such an awful crime as the sharpest evidence of the depth of demoralization to which we had attained before the revolution which must be impending on us as its necessary result. There is enough of Buncombe receptions, but of real appreciation of true heroism how much do we see?

Two men return to New York to-night who are in the truest use of the words heroes.

These are the Surgeons of the Eighth New York Regiment, whose officers deserted, in many cases, before coming under fire. They were in the Sudley church; they arrived at it amidst the hottest rush of the mad panic, but stopped to aid in taking care of the wounded, who had been placed for shelter in it. They were warned that the enemy was approaching; they heard the cry that the enemy was bayoneting the wounded and giving no quarter; they were advised to leave by a United States regular officer, who told them that the enemy would undoubtedly shell the church on the supposition that it would be held as a defensive position as long as possible by our troops covering the retreat. This conviction was so strong with those who left last, as you will remember, that it was generally reported that the church was shelled, and I have heard a man declare that he saw it shelled. But these surgeons said, simply, "We cannot leave these bleeding men; it is our business to take care of them, and take care of them we must, no matter what comes of us." They unquestionably made up their minds to die, and momentarily expected the falling of the first shell among them, until, while in the midst of an amputation, there was a rush of cavalry, and a horseman's pistol was pushed in at the door, and a hoarse voice asked, "Do you surrender?"

"Certainly," answered Swift, tying an artery, and not so much as raising his head from his business.

Would to God we had a few score such men as Captains, and would to God we had a General who would blow from the cannon's mouth such sham officers as those who played soldier with the Fire Zouaves and the Eighth, and such vermin as those of Varian's."—*N. Y. Times.*

---

*Chlorate of Potash Again.*—Some of the correspondents of the medical journals are raising the question of priority in the use of chlorate of potash in mercurial ptyalism, etc. The single fact is, it is an old remedy revived and, as usual, amplified and glorified. We have known it as a common remedy for mercurial salivation, stomatitis materna, scarlatina anginosa, etc., etc., for more than twenty years, and as far back as we can recollect in medicine it was by no means considered a new remedy. The priority does not rest with this generation.

